# The unique architecture and function of cellulose-interacting proteins in oomycetes revealed by genomic and structural analyses

**DOI:** 10.1186/1471-2164-13-605

**Published:** 2012-11-09

**Authors:** Mathieu Larroque, Roland Barriot, Arnaud Bottin, Annick Barre, Pierre Rougé, Bernard Dumas, Elodie Gaulin

**Affiliations:** 1 Université de Toulouse, UPS, Laboratoire de Recherche en Sciences Végétales, 24 chemin de Borde Rouge, BP42617, Auzeville, Castanet-Tolosan, F-31326, France; 2 CNRS, Laboratoire de Recherche en Sciences Végétales, 24 chemin de Borde Rouge, BP42617, Auzeville, Castanet-Tolosan F-31326, France; 3 Université de Toulouse, UPS, Laboratoire de Microbiologie et Génétique Moléculaire, Toulouse F-31000, France; 4 Centre National de la Recherche Scientifique; LMGM, Toulouse F-31000, France; 5 Present address: Université de Toulouse, UPS, Laboratoire PHARMA-DEV IRD UMR 152, 35 Chemin des Maraîchers, Toulouse 31400, France

**Keywords:** Cellulose, Oomycete, Lectin, Immunity, Plant, Adhesion, Fungi

## Abstract

**Background:**

Oomycetes are fungal-like microorganisms evolutionary distinct from true fungi, belonging to the Stramenopile lineage and comprising major plant pathogens. Both oomycetes and fungi express proteins able to interact with cellulose, a major component of plant and oomycete cell walls, through the presence of carbohydrate-binding module belonging to the family 1 (CBM1). Fungal CBM1-containing proteins were implicated in cellulose degradation whereas in oomycetes, the Cellulose Binding Elicitor Lectin (CBEL), a well-characterized CBM1-protein from *Phytophthora parasitica,* was implicated in cell wall integrity, adhesion to cellulosic substrates and induction of plant immunity.

**Results:**

To extend our knowledge on CBM1-containing proteins in oomycetes, we have conducted a comprehensive analysis on 60 fungi and 7 oomycetes genomes leading to the identification of 518 CBM1-containing proteins. In plant-interacting microorganisms, the larger number of CBM1-protein coding genes is expressed by necrotroph and hemibiotrophic pathogens, whereas a strong reduction of these genes is observed in symbionts and biotrophs. In fungi, more than 70% of CBM1-containing proteins correspond to enzymatic proteins in which CBM1 is associated with a catalytic unit involved in cellulose degradation. In oomycetes more than 90% of proteins are similar to CBEL in which CBM1 is associated with a non-catalytic PAN/Apple domain, known to interact with specific carbohydrates or proteins. Distinct Stramenopile genomes like diatoms and brown algae are devoid of CBM1 coding genes. A CBM1-PAN/Apple association 3D structural modeling was built allowing the identification of amino acid residues interacting with cellulose and suggesting the putative interaction of the PAN/Apple domain with another type of glucan. By Surface Plasmon Resonance experiments, we showed that CBEL binds to glycoproteins through galactose or N-acetyl-galactosamine motifs.

**Conclusions:**

This study provides insight into the evolution and biological roles of CBM1-containing proteins from oomycetes. We show that while CBM1s from fungi and oomycetes are similar, they team up with different protein domains, either in proteins implicated in the degradation of plant cell wall components in the case of fungi or in proteins involved in adhesion to polysaccharidic substrates in the case of oomycetes. This work highlighted the unique role and evolution of CBM1 proteins in oomycete among the Stramenopile lineage.

## Background

Carbohydrate-binding modules (CBM) are protein domains that recognize and bind to oligo- and polysaccharide ligands. Within the Carbohydrate-Active Enzyme Database (CAZy; CAZyme database, http://www.cazy.org, [1]), CBMs are divided into 64 families, based on amino acid sequence similarity, and members of each family display common ligand specificity. Regarding reported specificities, characterized CBMs have been found to bind to crystalline or non-crystalline cellulose, chitin, β-1,3-glucans, β-1,3-1,4-mixed linkage glucans, xylan, mannan, galactan or starch, while others behave like lectins, binding to a variety of cell-surface glycans [2].

As protein domains, one or more CBMs are generally associated with other protein domains, typically glycosyl hydrolase (GH) modules, and can be localized at either the N or C-termini of proteins, although proteins formed exclusively of single CBMs have been described [3,4]. Many CBMs have been biochemically characterized and structural data are now available providing insight into structure/function relations (for review see [2]). Since it is known that many CBMs are able to bind to polysaccharides, it is thought that when these are attached to catalytic domains their presence ensures intimate contact with the substrate and thus potentiated catalysis [5-7]. Moreover, it has been postulated that some CBMs might possess the ability to locally destruct polysaccharide structure (e.g. lower crystallinity in cellulose), thus improving enzyme accessibility [8].

Family 1 CBMs are small modules composed of 32 to 36 amino acids and are known as a “fungal CBM family”, because they were first detected in fungal cellulases and are exclusively produced by eukaryotes. The first characterized CBM1 was the cellobiohydrolase I from *Trichoderma reesei*[9]. Since then, numerous CBM1 proteins from various fungi have been reported [10-12]. The role of CBM1 in cellulases has been studied by separating the catalytic domain from its CBM, thus facilitating the study of the activity of the isolated catalytic domain on one hand and, on the other hand, the binding ability of the CBM. Data acquired in this way has indicated that CBM1 binds strongly to crystalline cellulose and that its presence is required for full activity of the enzyme against the insoluble polysaccharide [6,13,14]. A structural study of CBM1 from *T. reesei* cellobiohydrolase I have shown that overall architecture forms a wedge shape that is formed by irregular antiparallel triple-stranded β-sheet, which is stabilized by 2 disulfide bridges [15]. A flat face of the wedge bears three aligned aromatic residues (Y5, Y31 and Y32) that, along with polar residues (Q7 and N29), appear to form the cellulose binding interface [16,17]. This is corroborated by the fact that the removal of any of these residues reduces the ability of the enzyme to degrade crystalline cellulose [17]. Nevertheless, the role of CBM1 at the molecular level is not fully characterized.

Interestingly, CBM1-containing proteins have also been identified in fungal-like organisms called oomycetes [18]. Like fungi, oomycetes are ubiquitous in marine, freshwater and terrestrial environments [19]. They have similar modes of nutrition and ecological roles to true fungi and form tip-growing branching hyphae. Oomycetes were initially classified within the kingdom of Fungi, but molecular phylogenetic studies have now firmly established the distinct taxonomic positions of true fungi and oomycetes. Oomycetes belong to the Kingdom Stramenipila, which includes diatoms, chromophyte algae, and other heterokont protists [20-22]. Numerous oomycete species are plant pathogens, such as the causal agent of potato blight *Phytophthora infestans* or the sudden oak death pathogen *Phytophthora ramorum*. Features characterizing oomycetes are usually based on biochemical studies focused on *Phytophthora sp.* and particularly, the presence of cellulose rather than chitin in their cell wall. However the presence of either chitin or chitosaccharides was observed in the Saprolegniale oomycetes *Saprolegnia monoica* and *Aphanomyces euteiches*, where these compounds play an essential structural role [23,24].

The first oomycetal CBM1- protein described is the cellulose-binding elicitor lectin (CBEL) from *Phytophthora parasitica*, the causal agent of tobacco black shank disease [25]. This non-enzymatic cell wall glycoprotein harbors two CBM1s associated to two PAN/Apple modules known to interact with polysaccharides or proteins [26,27]. Knockdown *P. parasitica*-CBEL transformants are affected in cell wall polysaccharide deposition and adhesion to cellulosic substrates, including plant cell walls [28]. In addition to structural and adhesive roles, CBEL also induces plant defense responses, such as the production of reactive oxygen species, cytosolic calcium variation, expression of PR-proteins, and necrosis in several plant species [28,29]. The mutation of a recombinant CBEL has revealed that functional CBM1 is required for the full elicitor activity of CBEL [30]. Moreover, it has been suggested that interaction of CBM1 module with the plant cellulose microfibrils is perceived by plant cells as a warning signal [30,31]. Similar results have been obtained with a fungal CBM1 from *T. reesei* suggesting that plants are able to perceive oomycetal as well as well fungal CBM1s [32].

The discovery of CBM1-containing proteins in oomycetes has raised the question of their origin in a lineage distantly related to fungi. It has been recently suggested that some genes encoding proteins involved in the breakdown of plant cell wall components have been acquired by oomycetes from fungi through horizontal gene transfer [33]. However, CBM1-containing proteins were not detected in this analysis.

To better understand the origin, evolution and biological role of CBM1-containing proteins in oomycetes, we have performed data mining of fungal and oomycetal genomes and compared the protein organizations of different CBM1-containing proteins. In this way, we have revealed that oomycete-unique association of CBM1 with PAN/Apple domains.

Moreover, using CBEL from *P. parasitica*, which was shown to be *a bona fide* cellulose-binding protein, we propose a model structure of an oomycetal CBM1 and a role for the PAN/Apple domain in binding of the protein to additional carbohydrates. Accordingly, we present experimental evidence for a galactose or N-acetylgalactosamine-specific lectin activity associated with CBEL. Taken together, the results suggest that oomycetal CBM1-containing proteins have an ancient origin in the oomycete lineage and are involved in specific roles including adhesion to self and non-self components rather than in substrate degradation.

## Results

### Establishment of a comprehensive repertoire of CBM1-containing proteins in fungi and oomycetes

**Figure 1:**
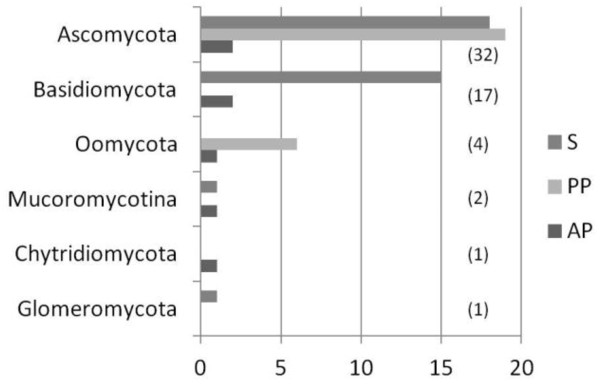
**Number of fungal and oomycetes species used in the analysis.** Species are classified as Saprobe (S), Plant parasite (PP), Animal parasite (AP). Number of genus represented in each lineage is indicated in brackets. The names of fungal and oomycete species used in this study are indicated in the Additional file 1:Table S1.

CBM1-containing proteins were collected from the CAZy database [1] and curated for Stramenopiles sequences which were further retrieved by mining predicted proteome on dedicated databases (Additional file 1: Table S1). Seven oomycete genomes were mined for CBM1 (IPR000254) containing sequences. The oomycete species studied are characterized by different lifestyles, namely the obligate biotrophic species *Hyaloperonospora arabidopsidis* and *Albugo laibachii*, four hemibiotrophic *Phytophthora* species (*P. infestans, P. sojae, P. ramorum, P. capsici*) and the necrotroph *Pythium ultimum*. Sequences from various fungal databases, absent in CAZy (i,e *Chaetomium globosum*), were added. Overall a set of 60 fully sequenced fungi representing the major evolutionary lineages within the fungal kingdom were included in the analysis (Figure 1). Sequences were collected to build a representative set spanning the diversity of fungi and oomycetes. In total 518 sequences were selected for further analyses.

**Figure 2:**
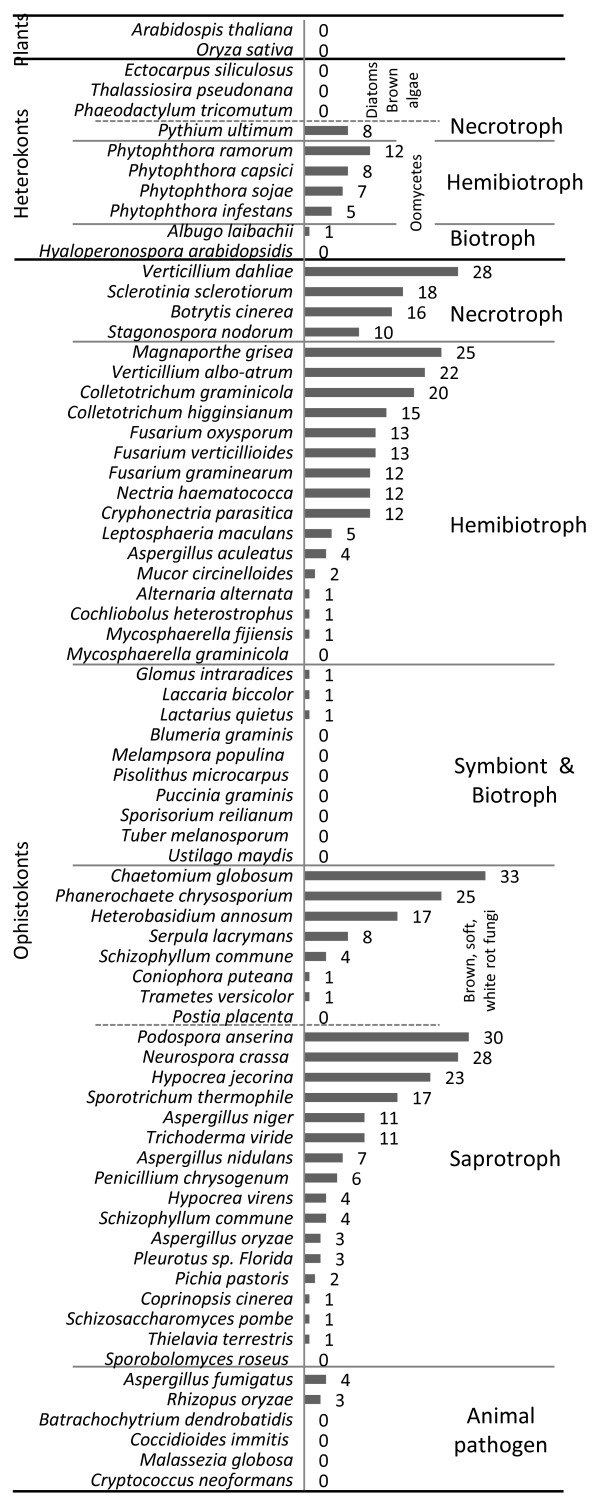
**Distribution of CBM1-containing proteins in fungi and oomycetes.** Proteomes were screened for the presence of CBM1 domain (IPR00254) thanks to the InterProScan software. The number of putative CBM1-containing proteins per species is reported on the graphic. Microorganisms are classified based on their life style as indicated. Genomes of diatoms (*Phaeodactylum tricomutum, Thalassiosira pseudonana*), brown algae (*Ectocarpus siliculosus*) and plants (*A. thaliana, Oryza sativa*) are included in the analysis.

The survey of CBM1 revealed the presence of at least one CBM1-protein in 43 of the 67 (77%) completed fungal/oomycete genome sequences examined. The total numbers of CBM1-containing proteins per sequenced fungal/oomycete species classified by life style (saprotroph, necrotroph, hemibiotroph, symbiont and animal pathogen) is shown in Figure 2. *Chaetomium globosum* as well as two other saprobes, the white rot fungus *Phanerochaete chrysosporium* and the dung fungus *Podospora anserina* encode the highest number of predicted CBM1-containing protein coding genes among sequenced fungal genomes. A closer look shows that most fungi that harbor necrotrophic or hemibiotrophic infection strategy (e.g. *Verticillium dahliae, Magnaporthe grisea*), have the highest number of CBM1-protein coding genes per genome (>10 up to 28 CBM1 copy number). Interestingly, none or a single CBM1-protein gene is detected in biotrophic plant pathogen (e.g. *Ustilago maydis, Puccinia graminis, Melampsora populina*) and symbionts (e.g. *Glomus intraradices, Laccaria bicolor, Tuber melanosporum*). Thus, the expansion of CBM1-encoding genes in phytopathogenic fungi is correlated with hemibiotrophic or necrotrophic lifestyles. However, genes encoding CBM1-containing proteins are not restricted to fungal plant pathogens. Indeed, the same number of genes encoding CBM1-containing proteins is present in *Aspergillus fumigatus* and *A. aculeatus*, the former being a human pathogen and the latter a plant pathogen. Likewise, the opportunist human pathogen *Rhizopus oryzea* also displays genes encoding CBM1-containing proteins while genomes of other animal pathogens, including the amphibian pathogen *Batrachochytrium dendrobatidis*, lack detectable genes encoding cellulose-binding proteins.

Rather like true fungi, necrotrophic and hemibiotrophic oomycetes contain more genes encoding CBM1-containing proteins (up to 12) than biotroph species (*A. laibachi, H. arabidopsidis*). To complete this study, the genomes of *Arabidopsis thaliana* and *Oryza sativa*, and those of the closet phylogenic cousins of oomycetes, the diatoms *Thalassiosira pseudonana, Phaeodactylum tricomutum* and the brown algae *Ectocarpus siliculosus*, which also belong to the heterokont lineage, were screened for CBM1-encoding sequences. However, no CBM1-encoding sequences were detected in these organisms.

### Fungal and oomycetal CBM1-containing proteins display distinct domain associations

**Figure 3:**
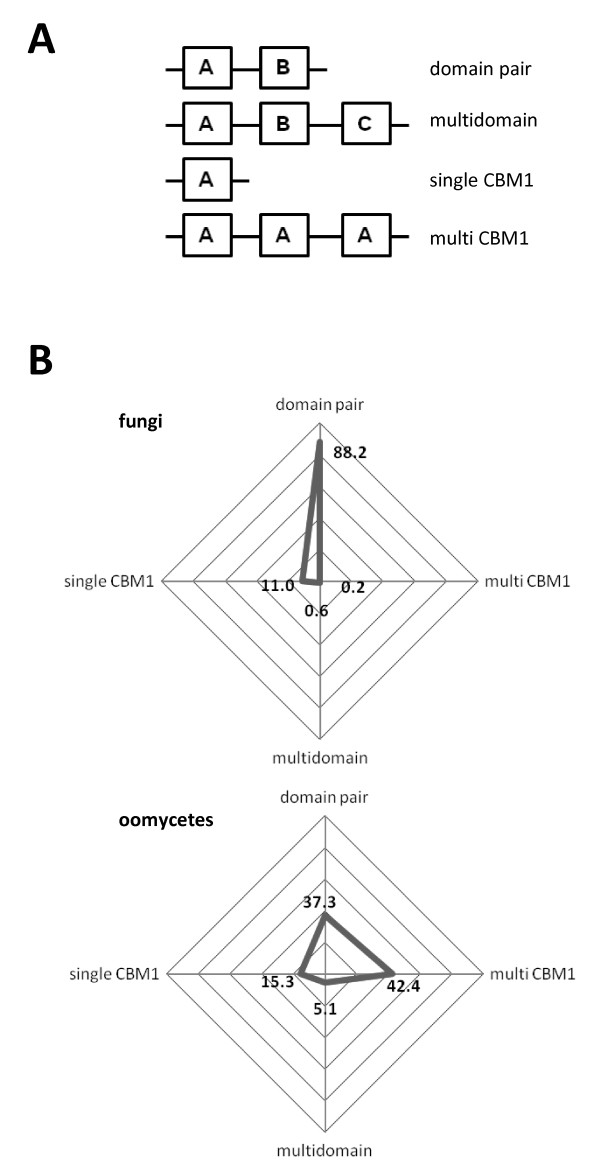
**Distribution of CBM1 domain association categories in fungi and oomycetes.A**) Four categories are defined based on the individual domains position and frequency of CBM1. BoxA: CBM1, BoxB and BoxC: could be either catalytic or non catalytic domain. **B**) Radar graphic displaying the overall distribution of the different domain association categories in fungi and oomycetes. Numbers indicated the value in percent of each category.

The domain architecture of CBM1-containing proteins was investigated using the boundaries defined in Pfam/Smart annotations. Domain architectures were assigned to 4 categories: i) "domain pair" which are sequences that contain a CBM1 and one other domain, ii) "multi-domain", which are sequences that contain one CBM1 and two other domains, iii) "single CBM1", which are sequences that are composed of a single isolated CBM1 and iv) "multi-CBM1", which are sequences that display more than one CBM1 (Figure 3A). Among eukaryotes, fungal proteomes display more than 50% of proteins can be classed as ‘single domain’ [34]. Regarding CBM1 associations, the ‘single domain’ category is somewhat under-represented and pertains to roughly ~15% of the fungal and oomycetal CBM1-containing proteins (Figure 3B). In contrast, 88% and 37% of fungal and oomycetal CBM1-containing proteins respectively possess ‘domain pair’ architecture. Moreover, the ‘multi CBM1’ architecture is more widespread in oomycetes (42%) than in fungi (0.2%).

In fungi, 89% of the ‘domain pair’ category are proteins that contain a CBM1 appended to a catalytic domain belonging to the GH superfamily and 11% correspond to a CBM1 appended to non-catalytic modules (chitin-binding module, carbohydrate-binding module family 4, fucose lectin, unknown (DUF) or wide functions (BNR/Asp-box repeats, see Additional file 2: Table S2 for IPR number of corresponding domain). In sharp contrast, in oomycetes 90% of the proteins in the ‘domain pair’ category are characterized by a CBM1 associated with a non-catalytic domain, corresponding to the PAN/Apple module, which are known to display protein-carbohydrate or protein-protein functions. Using SignalP 3.0 [35], it was found that 91 % of the proteins are predicted to contain a signal peptide, indicating that they are probably secreted. Overall, the large-scale systematic analysis of domain architecture reported here clearly revealed the distinct architecture of fungal and oomycetal CBM1-containing proteins.

**Figure 4:**
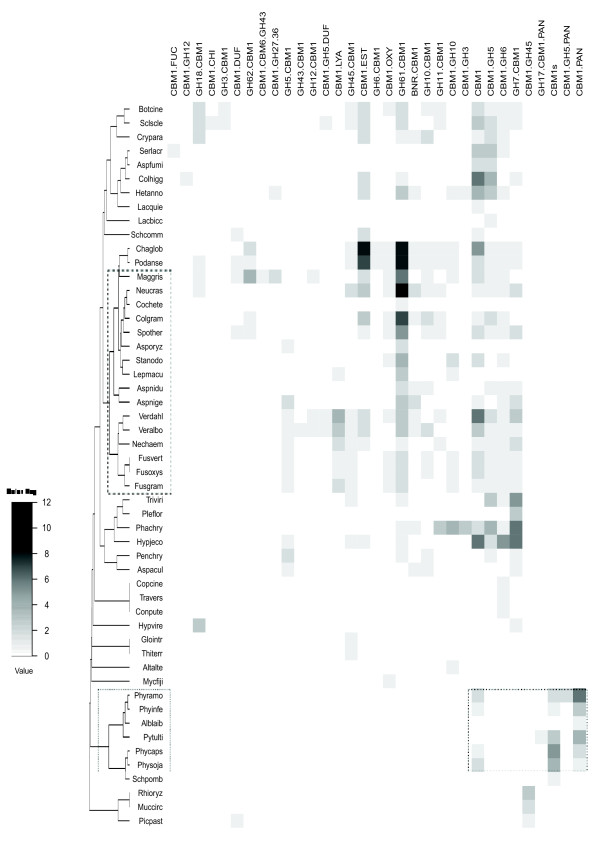
**Hierarchical clustering based on CBM1 domain combinations in fungi and oomycete genomes.** Rows correspond to species and columns to architecture of CBM1-containing proteins. Grey intensity on the heat map represents the number of proteins found for a domain combination in a microorganism. The oomycete cluster is indicated as a black line and fungal pathogens as dashed box. Alias used for the species names are indicated in Additional file 1: Table S 1.

Taking our study further, we compared the architecture distribution between the different fully-sequenced organisms. The abundance of domain associations per genome was computed and hierarchical clustering was used to assemble similar domain associations. An overview of the heat map (Figure 4, Additional file 2: Table S2) reveals a distinct distribution of domain combination in fungal and oomycetal proteins. A large proportion of fungal proteins, encompassing 70% of fungal plant pathogens, is characterized by CBM1s associated with catalytic domains involved in cellulose degradation. This includes fungal cellobiohydrolases (GH6.CBM1, GH7.CBM1) and β-glucosidases, exemplified by the CBM1.GH3 association. Interestingly, a large number of fungal CBM1-containing proteins are characterized by the GH61.CBM1 association. The GH61 family was originally described as endo-1,4-β-D-glucanases displaying weak activity, but recent studies have shown that GH61 members are in fact copper-dependent oxidases, that promote lignocellulosic substrate degradation [36,37]. Within this main group, one can observe a subgroup exclusively constituted by fungal plant pathogens that cause plant wilting symptoms (*Verticillium sp. and Fusarium sp*.), which are provoked by the colonization and proliferation of the microorganism in the xylem vessels of the host. In this small cluster, proteins predicted to degrade pectin (polysaccharide lyase families PL1 and PL3 appended to a C-ter CBM1) are also found.

Oomycetes species are clustered in one, unique group presenting two main characteristics. First, catalytic domains classified in the CAZyme database are not detected, except those of the GH5 family. Second, most of CBM1-containing proteins are organized as multiple CBM1 modules, which are either standalone proteins or are appended to PAN/Apple domains. Therefore, to investigate whether the absence of GH domains in oomycete CBM1-containing proteins reflects a general scarcity of glycosyl-hydrolases genes in their genome, the number of GH genes per genome was calculated. As shown in Table 1, oomycete genomes contain a large set of glycosyl-hydrolases genes (>100 gene models/genome), except *A. laibachii* (<50). Thus the rarity in oomycetes of GH.CBM1 associations, coupled to an enrichment of CBM1.PAN/Apple associations, supports the hypothesis that specific functions can be attributed to CBM1-containing proteins from these microorganisms and bears witness to a distinct evolutionary history compared to fungi.

**Table 1:** Occurrence of CBM1 in glycosyl-hydrolases in fully sequenced oomycete genomes and selected fungal plant pathogens

**Number of glycosyl-hydrolases predicted genes**				
		**Total**	**with CBM1**	**(%)**
**Oomycetes**	*A. laibachii*	44	0	none
	*P. infestans*	157	0	none
	*P. ramorum*	114	2	1.8
	*P. sojae*	125	0	none
	*P. ultimum*	85	1	1.2
**Fungi**	*B. cinerea*	118	11	9.3
	*F. oxysporum*	368	9	2.4
	*M. grisea*	232	19	8.2
	*V. dahliae*	281	14	5.0

Data were collected from *A. laibachii*[69], *Phytophthora sp*[70]*, P. ultimum*[71], *B. cinerea*[72], *F. oxysporum, V. dahliae* and *M. grisea*[73].

### Sequence and structural analysis of oomycetal CBM1-containing proteins

**Figure 5:**
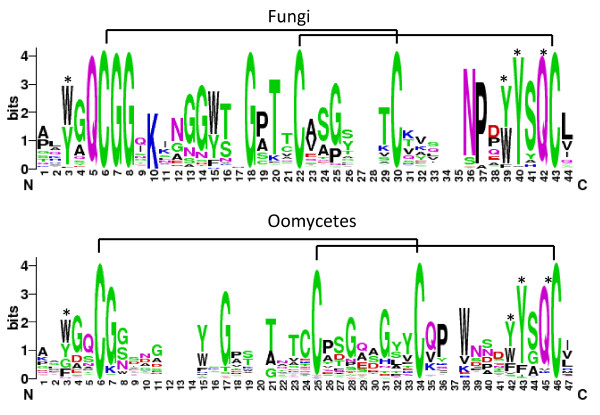
**Weblogos built from fungal and oomycetal CBM1 sequences.** The height of the residues represents their conservation among the peptide sequences. Cysteine residues engaged in disulphide bonds are materialized as black lines. The glutamine and aromatic residues that are known in fungi to be important for interaction with crystalline cellulose are shown by asterisks. Similarly these residues susceptible in oomycetes to interact with the carbohydrate substrate are depicted with asterisks.

In order to identify residues that are conserved in both fungal and oomycetal CBM1 domains, the CBM1 amino acid sequences of the 518 proteins were extracted and visually compared using WebLogo [38]. As shown in Figure 5, cysteine residues known to participate in the folding of fungal CBM1s [18] appeared at conserved positions in the oomycete sequences, though an additional cysteine residue was also detected (C^24^). The conserved doublet of glycine residues found in the N-termini of fungal CBM1s is not conserved in oomycetes but the aromatic amino residues ([WY]^3^, [WY]^39^, Y^40^) and the polar residue glutamine (Q^42^) that are known binding determinants in fungal CBM1s [4,6,17] were frequently identified in the oomycete sequences. Nevertheless, tryptophan and tyrosine residues in these motifs were often replaced by phenylalanine, suggesting greater diversity among the cellulose-binding determinants in oomycetes than in fungi.

**Figure 6:**
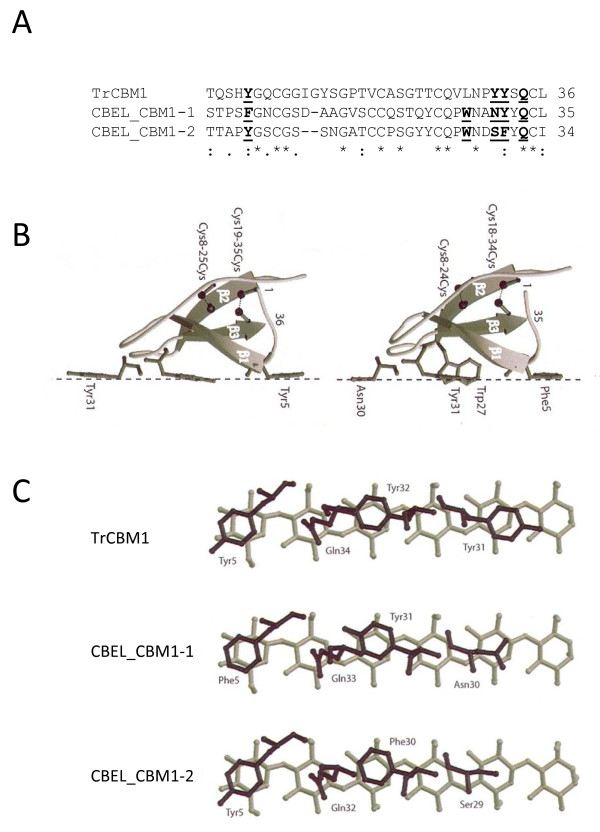
**Modeling of the CBM1s from the CBEL protein.A**) Multiple sequence alignment of CBEL_CBM1-1, CBEL_CBM1-2 with the CBM1 of cellobiohydrolase I from *Trichoderma reesei* (TrCBM1, Uniprot/KB accession number: P62694). Amino residues putatively important for binding to crystalline cellulose are in bold and underlined. **B**) Comparison of the lateral faces of the ribbon diagrams of TrCBM1 (left) and CBEL_CBM1-1 (right). The strands of the β-sheet are numbered β1, β2 and β3 from the N- to the C-terminus. The aromatic residues involved in the binding to cellulose are displayed in grey ball-and-sticks. The Asn (replaced by Ser in CBEL_CBM1-2) and Trp residues susceptible to interact with cellulose are similarly displayed in grey ball-and-sticks. The four Cys residues linked to disulphide bonds (dashed lines) are displayed in black ball-and-sticks and numbered. **C**). Precise view of the binding model of CBM1s from TrCBM1 and CBEL. Only the aromatic residues (Phe and Tyr) stacking on the pyranose ring of the glucose units and close hydrophilic residues (Gln, Asn and Ser) susceptible to create hydrogen bonds with the hydroxyls of the glucose units, are displayed in black ball-and-sticks. Only six β1,4-linked glucose units are drawn in grey ball-and-sticks. Cartoons are drawn with Molscript [67], and rendered with Raster3D [68]

To detect structural specificities of oomycete domains, a modeling approach was undertaken using the best characterized oomycetal cellulose-interacting protein, CBEL. This protein is composed of a single polypeptide chain of 266 amino acids which starts with a 20 residue long signal peptide and then is built from the two symmetric N- and C- terminal parts, each resulting from the association of a CBM1 and a PAN/Apple domain (CBM1-1:21–54 + PAN1: 55–133; CBM1-2: 158–190 + PAN2: 191–268) separated by a hinge region (134–157) that is rich in proline and threonine residues. The rather high percentage of identity (~37%) and similarity (~60%) between the *T. reseei* cellobiohydrolase I CBM1 and the CBM1s of CBEL (Figure 6A) meant that the CBM1s exhibited very similar HCA plots (data not shown), thus allowing the accurate prediction of the three β-sheet strands in both CBEL_CBM1-1 and CBEL_CBM1-2. In addition the four cysteine residues forming two disulphide bridges in TrCBM1 are strictly conserved in the CBEL_CBM1-1 as shown by the alignment of the domains (Figure 6A). Accordingly, the predicted three-dimensional models of CBEL_CBM1-1 and CBEL_CBM1-2, built from the NMR-coordinates of TrCBM1 (RCSB PDB code 1CBH), exhibited a very similar fold (Figure 6B). They consist of three anti-parallel strands of β-sheet (β1, β2 and β3) interconnected by loops. Two disulphide bonds (C^8^-C^24^ and C^18^-C^34^ in CBM1, C^8^-C^23^ and C^17^-C^33^ in CBM1-2) contribute to the stability of folding, and especially to the stability of the stands (β1 and β3 and loop (loop connecting strands (β2 to β3) which contains the exposed aromatic residues involved in cellulose binding. Both CBM1s contain two exposed aromatic residues F^5^ and Y^31^ in CBM1-1 and Y^5^ and F^30^ in CBM1-2 that are homologous to Y^5^ and Y^32^ of TrCBM1 and are thus suitability positioned to stack onto cellulose chains (Figure 6B and C). An additional Q^34^ residue is also known to participate in the binding of TrCBM1 to cellopentaose and cellohexose [39]. This residue is conserved in CBEL (Q^33^ in CBEL_CBM1-1, Q^32^ in CBEL_CBM1-2), suggesting that it could participate in binding to crystalline cellulose (Figure 6C).

**Figure 7:**
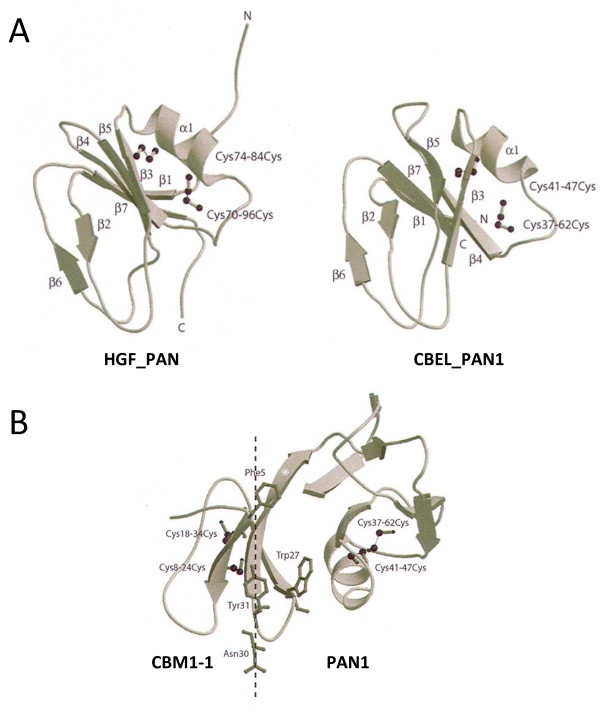
**Stereo ribbon representation of PAN and CBM1-PAN1 from CBEL bound to cellulose.A**) Comparison of the ribbon diagrams of domains HGF_PAN (left) and CBEL_PAN1-1 (right). The α-helix (α1) and strands of b-sheets (β1-β2) are numbered from the N- to the C- terminus. The cysteine residues linked by disulphide bonds (dashed lines) are displayed in black ball-and-sticks and numbered. **B**) The cysteine residues linked by disulphide bonds (dashed lines) are displayed in black ball-and-sticks and numbered. The extended strand of β-sheet interconnecting the two domains is indicated by an asterisk. The aromatic residues of CBM1 interacting with cellulose are in grey ball-and-sticks and labelled. The figures are drawn using Molscript [67], and Raster3D [68].

Although the PAN/Apple domains of CBEL share low identity (~20%) and similarity (~36%) with the PAN/Apple domain of the human hepatocyte growth factor (HGF), their HCA plots suggested a very similar structural organization (data not shown). All PAN/Apple domains consist of three and four strands of β−sheets separated by a short α-helical segment. The four cysteine residues linked by two disulphide bridges occurring in the HGF PAN/Apple domain are conserved in both CBEL_PAN/Apple domains. The three-dimensional models of CBEL_PAN1 and CBEL_PAN2 domains built from the NMR-coordinates of the N-terminal human HGF PAN domain (RCSB PDB code 2HGF) consist of a central five-stranded antiparallel β−sheet associated to a short α-helical segment and to two loops containing a short strand of β−sheet (Figure 7A). In the HGF-PAN/Apple domain, the α−helix is linked to two antiparallel strands of β−sheets by an extended loop to form a hairpin-loop region stabilized by two disulphide bridges. A similar harpin-region stabilized by the disulphide bridges C^8^-C^24^ and C^18^-C^34^ (CBM1-2) or C^8^-C^23^ and C^17^-C^33^ (CBM1-2) also occurs in CBEL. However, the hairpin-loop region is shortened by the deletion of four amino acid residues corresponding to the ^80^LPFT^83^ motif of the HGF-PAN/Apple domain. The carbohydrate-binding sites of plant lectins usually consist of shallow depressions resulting from the confluence of exposed loops containing hydrophilic and acidic (Asp or Glu) residues that anchor a sugar residue through a network of hydrogen bonds [40]. In addition, an aromatic residue (*i.e.* F or Y) completes the interaction by stacking against the pyranose ring of the sugar. The two exposed loops ^18^Asn-Val-Asp-**Phe-**Arg-Gly-Asp-Asp^35^ of PAN1 or ^18^Asp-Lys-Asp-**Tyr**-Arg-Gly-Asn-Asp^25^ of PAN2, and ^66^Ser-Gly-Thr-Gly-Thr-Arg-Thr^72^ of PAN1 or ^66^Ser-Ala-Ala-Gly-Thr-Ala-Thr^72^ of PAN2, fulfill these structural requirements and thereby could act as the carbohydrate-binding sites responsible for the hemagglutinating activity of CBEL [18,41].

Six amino acid residues (NYYQCL for CBM1-1/PAN1, SFYQCI for CBM1-2/PAN2) that form the transition between the CBM1s and PAN domains in CBEL correspond to a strand of β−sheet (YQCL and YQCI in CBEL_CBM1-1 and CBEL_CBM1-2 respectively). The first strand of β−sheet (LDLPA in CBEL-PAN1 and IQPPA in CBEL-PAN2) of the CBEL-PAN/Apple domains also contains the ultimate leucine or isoleucine residue of the β−strand of the CBEL-CBM1s. This suggests that a continuous strand of β−sheet (YQCLDLPA and YQCIQPPA) interconnects the CBM1 and PAN domains in the CBEL molecule. Using this information, a tentative three-dimensional model was built for the CBM1-1/PAN1 N-terminal part of CBEL (Figure 7B). Although speculative, this model readily fulfills geometric constraints (>70% of residues occur in the allowed areas of the Ramachandran plot and >90% in the generously allowed area) and reasonably accounts for both the associated cellulose-binding and carbohydrate-binding properties previously reported for CBEL [18,41]. A very similar model was obtained for the CBM1-2/PAN2 C-terminal part of CBEL (result not shown).

### Surface Plasmon resonance analysis of CBEL lectin activity

**Figure 8:**
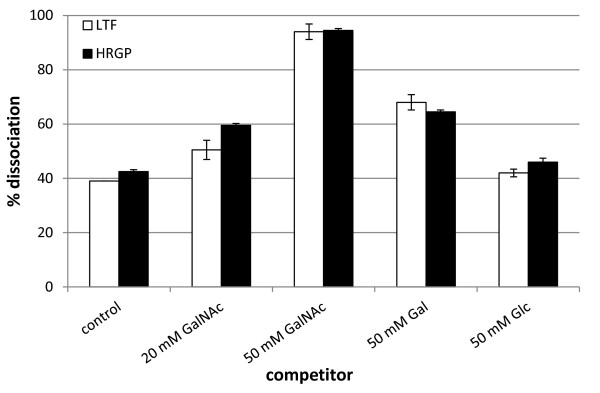
**Surface Plasmon Resonance analysis of CBEL-glycoproteins interactions.** Human lactotransferin (LTF) or melon hydroxyprolin-rich glycoprotein (HRGP) were passed over the CBEL chip surface at concentrations of 25 or 100 μg/ml respectively. Then, either HBS-Ep buffer alone (control) or monosaccharide solutions (GalNAc, Gal, Glc) were injected during the dissociation phase, 50 or 94 seconds after the end of LTF or HRGP injection respectively. The percentage of protein complex dissociation was measured 385 seconds after the beginning of buffer or monosaccharide injection. Data are the means of the values recorded on the two channels of the Biacore X flow cell ± SD.

In initial studies, the lectin activity of CBEL was demonstrated by a hemagglutination test [18]. To get further insight into CBEL lectin activity and confirm the presence of the carbohydrate binding site inferred from molecular modelling, Surface Plasmon Resonance (SPR) technology was used. Since the detection of molecular interactions using this technology is highly dependent on the molecular weight of the analytes [42], rather that attempting the direct measurement of the interaction of CBEL with various low molecular weight single sugars, we decided to check whether single sugars could interfere with the binding of large glycoproteins to CBEL. Screening of a collection of various pure glycoproteins allowed to detect the association of CBEL with human lactotransferrin and with a melon hydroxyproline-rich glycoprotein [43,44], both glycoproteins sharing the fact that their glycan moiety contain galactose residues. Subsequent assays showed that the respective protein complexes were strongly destabilized by *N*-acetylgalactosamine (Figure 8). Galactose also showed some activity in this assay, though less pronounced. This data confirms the lectin activity of CBEL and suggests that it is involved in binding to polysaccharides or glycoproteins through the interaction with N-acetylgalactosamine or galactose residues.

## Discussion

In this work, a comprehensive set of fungal and oomycetal CBM1-containing proteins was described on the basis of CBM1 detection, and the domain organization of proteins was predicted. CBM1-containing protein genes were found in the vast majority of the analyzed genomes, and their number was clearly related to the lifestyle of the microorganisms. Fungal and oomycetal saprotrophs, necrotrophs and hemibiotrophs express the largest number of CBM1-encoding genes whereas very few, if any, are found in biotrophs. This result is correlated with a dramatic reduction of proteins interacting with plant cell wall components in fungal or oomycete biotrophs [45,46]. This situation could be explained by the induction of plant defense by plant cell wall-interacting proteins, which could be detrimental to the biotrophic lifestyle. In the case of oomycetes, this interpretation could be extended to non-enzymatic proteins such as CBEL, which has been shown to be a potent elicitor of plant defenses [18,29,30]. While necrotroph and hemibiotroph oomycetes encode 5 to 12 cellulose-interacting proteins, only one was found in the biotroph *Albugo laibachii* and no unambiguous CBM1-encoding genes were detected in the *Hyaloperonospora arabidopsidis* genome.

We further observed that oomycetes express a unique combination of domains corresponding to an association of a CBM1 with a PAN/Apple module. This combination does not appear in other eukaryote including diatoms and brown algae. So far, no enzymatic activity has been associated with CBM1-PAN/Apple proteins, with the best characterized protein of this type being the *P. parasitica* CBEL. It has been shown that CBEL plays a major role in *Phytophthora* cell wall integrity, probably through interactions with the cellulose component of the oomycetal cell wall [28]. Thus, oomycete CBM1-containing proteins play multiple roles that target both the plant and the oomycete cell walls. However, this situation might be unique in the Stramenopile lineage, since no CBM1-containing proteins were detected in other stramenopiles with a cellulosic wall, such as *Ectocarpus silicolosus*[47]. Strikingly, while oomycetes potentially express a large set of glycosyl-hydrolases, only 2 genes were found to be associated with a CBM1, whereas in fungi CBM1 is almost exclusively associated with an enzymatic domain. This result probably bears witness to the distinct evolutionary history of CBM1 and plant cell wall degrading enzymes in fungi and oomycetes. This could be related to the specific functions of CBM1-containing proteins, which in oomycetes are involved in cell wall organization, whereas in fungi, CBM1-containing proteins only target plant components, since fungal cell walls do not contain cellulose.

While CBM1s from oomycetes share similarity with fungal CBM1s, they also display specific features, as revealed by sequence alignment and structural modeling of CBM1s from CBEL. This protein has been shown to bind crystalline cellulose and mutation of its CBM1s has revealed their role in cellulose binding [30]. The two CBM1s of CBEL, which both exhibit two well-exposed, spatially-aligned aromatic residues (F^5^ and Y^31^ of CBM1-1, Y^5^ and F^30^ of CBM1-2), readily account for the cellulose-binding properties displayed by both native and recombinant proteins [30]. These aromatic residues are homologous to the aromatic residues (Y^5^, Y^31^, Y^32^) that are cellulose-binding determinants in the C-terminal CBM1 of the *T. reesei* cellobiohydrolase I [16,17], and are thus suspected to fulfill a similar function. Indeed, mutational analysis of these residues revealed their important role in the binding of CBEL to cellulose and cell wall components [30]. An additional Q^34^ residue (conserved as Q^31^ and Q^32^ in the CBM1-1 and CBM1-2 of CBEL, respectively) also participates in the binding of the TrCBM1 to cellopentaose and cellohexaose [16]. This residue is conserved in CBEL_CBM1-1 (Q^34^) and CBEL_CBM1-2 (Q^33^), suggesting that it could also participate in conferring cellulose-binding ability to CBEL. Moreover, residue N^30^ of CBEL_CBM1-1 and S^29^ of CBEL_CBM1-2 are well exposed and spacially-aligned with the aromatic binding determinants, and thus could also participate in cellulose binding. This is also the case for W^27^ of CBEL_CBM1-1 and W^26^ of CBEL_CBM1-2, which is also located in the vicinity of the aromatic residues.

The occurrence of two PAN/Apple domains in CBEL offers an interesting example of the widespread distribution of a structural motif among proteins of very distinct origins [26,27,48,49]. Although the exact function of the PAN/Apple motif still remains questionable, its involvement in protein-protein and protein-carbohydrate interactions has been postulated from studies performed on plasminogen [50], prekallikrein [51] and on the human hepatocyte growth factor [26]. Interestingly, a PAN/Apple surface protein from the apicomplexan parasite *Toxoplasma gondii* has recently been shown to bind chondroitin sulfate, a sulfated glycosaminoglycan found attached to proteins as part of surface proteoglycans in animal cells [52]. One main constituent of the chondroitin polymer is N-acetylgalactosamine [53]. Accordingly SPR analysis suggests that CBEL is a *N*-acetylgalactosamine or galactose- binding lectin, two sugars that are frequently found as components of glycoprotein glycans. Therefore, it can be hypothesized that this lectin activity is mediated by the PAN/Apple domain.

The tentative three-dimensional model proposed for the molecular organization of the CBM1-PAN/Apple association suggests that there is no steric hindrance between the two domains, thus allowing CBEL to simultaneously display cellulose-binding and lectin activities. It has been shown that CBEL is localized both in the inner and outer cell wall layers of *P. parasitica*[25], a dual localization which is coherent with its molecular organization and its multiple functions [30]. Interaction of CBEL with a complex glycan, eventually bound to a cell wall polypeptide, could help its proper targeting and molecular docking to endogenous or exogenous cellulose microfibrils, and hence play roles both in cell wall scaffolding and in adhesion to exogenous cellulose. A lectin-based study has shown the presence of galactose and *N*-acetylgalactosamine residues at the cell surface of the oomycete *P. parasitica*[54]. Likewise, a proteomic analysis has shown that mucins, which are known to be highly glycosylated galactose- and *N*-acetylgalactosamine-containing proteins [55], form part of the *P. infestans* cell wall proteome [56]. This strongly suggests the presence of endogenous ligands for the anchoring of CBEL to the oomycete cell wall through its lectin activity. The fact that CBEL is formed from two repeated CBM1-PAN/Apple associations further reinforces the potential of CBEL as a versatile adhesin. In this context, the binding of CBEL to plant HRGPs could be of biological importance and should be further investigated.

The unique symmetric organization of CBEL provokes the question of the molecular evolution of this type of molecule. CBEL probably results from the duplication and fusion events of an ancestral gene, which itself results from the previous fusion of two genes encoding CBM1 and PAN/Apple domains respectively. So far, this particular combination of domains has only been found in oomycetes belonging to the Peronosporale lineage, since in distantly related oomycetes, such as *A. euteiches* or *S. parasitica*, CBM1s are associated with another type of interacting domain [57]. Further analysis of oomycete genomes will probably help to clarify the origin of CBM1-containing proteins. The recent identification of CBM1-encoding genes in the basal oomycete *Eurychasma dicksonii* (Gachon and Kim, personal communication), suggests an ancient origin of these proteins, probably related to their specific role in the oomycete cell wall and during interaction with their host.

## Conclusions

Using a genome mining approach to analyse fungal and oomycetal genomes, this study aimed at elucidating the origin and function of CBM1-containing proteins from oomycetes. Accordingly, we revealed a unique combination of domains in these organisms in which CBM1s are linked to PAN/Apple domains. This finding in combination with 3D structural modelling and Surface Plasmon Resonance analysis indicate that while CBM1s from fungi and oomycetes are similar, they are actually associated with different protein domains that confer quite different functions: while fungal CBM1s are combined to plant cell wall degradation enzymatic domains, those of oomycetes are associated with domains involved in adhesion to endogenous or exogenous ligands.

## Methods

### Data collection

Protein sequences were collected from the CAZy database [1], and then curated for oomycetes sequences. Oomycetes proteomes (*Phytophthora infestans, P. sojae, P. ramorum, P. capsici*, *Albugo laibachii*, *Hyaloperonospora parasitica*) were downloaded from the Broad Institute or the JGI, and submitted to an InterPro analysis to detect CBM1-containing proteins [58]. The recently sequenced fungal genomes, not yet referenced in the CAZYdatabase, were added after screening their proteome with the InterPro software to identify CBM1-containing proteins (e.g. *Chaetomium globosum*). Only sequences with E-values below 10^-6^ for PFAM or SMART domains corresponding to IPR000254 – Cellulose binding domain, fungal – were kept to minimize false positives. For a list of completely sequenced organisms that were used in this analysis see Additional file 1: Table S1.

### Domains architecture determination

All CBM1-containing proteins were submitted to a local InterProScan to identify other domains in the peptide sequences from the SMART and PFAM databases. Domains identified by both the SMART and PFAM databases were merged after checking their compatibility (location and InterPro identifiers equivalence) by adjusting domain boundaries to the largest domain. Overall, no domain inconsistencies were found between the two databases.

### Generation of protein architecture heatmaps

Previously determined protein domain architectures were summarized as follows to obtain more general architecture classes. When multiple CBM1 domains were found in the protein, the architecture is denoted CBM1s. Domains appearance on the sequence was reordered alphabetically except for glycosyl-hydrolase domains *i.e.*, both CBM1-LYA architecture (2 proteins) and LYA-CBM1 architecture (2 proteins) were set to CBM1-LYA whereas CBM1-GH5 architecture and GH5-CBM1 architecture were kept distinct. Multiple occurrences of BNR – bacterial neuramidase repeat – were simplified to only one occurrence *i.e.*, BNR-BNR-BNR-BNR-BNR-CBM1 was mapped to the BNR-CBM1 class. This was motivated by the fact that the number of BNR found in proteins varies from 5 to 8 occurrences and this domain is found in only 5 CBM1-containing proteins. After this generalization, there are 35 distinct classes of protein domain organization. These classes were used to build a contingency table of species and architecture classes. The rows correspond to species profiles: this provides for each species the number of proteins found exhibiting each domain organization. The columns correspond to protein architectures providing the number of proteins found in each species exhibiting such a domain organization. The species and architecture profiles were grouped by hierarchical clustering (average linkage using Euclidean distance) and the resulting classifications were used to draw a heat map in which cell intensities reflect the number of proteins found having a given architecture (column) in a given species (row).

### Molecular modeling of CBM1 and PAN domains from CBEL

The HCA (Hydrophobic Cluster Analysis [59]) was performed to delineate the conserved secondary structural features (strand of β-sheet and stretches of a-helix) along the amino acid sequence of CBEL by comparison with the CBM1 of the cellobiohydrolase I from *Trichoderma reesei*[15] and the PAN domain of the human hepatocyte growth factor (HGF, [60]) used as models. Multiple amino acid sequence alignments were carried out with CLUSTAL-W [61]. HCA plots were generated using the program drawhca of L. Canard ( http://mobyle.rpbs.univ-paris-diderot.fr/cgi-bin/portal.py?form=HCA). Molecular modeling of the CBM1s and PANs of CBEL was carried out on a Silicon Graphics O2 R10000 workstation using the program InsightII, Homolgy and Discover3 (Accelerys, San Diego CA, USA). The atomic coordinates of the CBM1 of the cellobiohydrolase I of *T. reesei* (RCSB Protein Data Bank code 1CBH) and the PAN domain of the human HGF (RCSB Protein Data Bank code 2HGF), were used to build the three-dimensional models of the homologous CBEL domains. Steric conflicts were corrected during the model building procedure using the rotamer library [62] and the search algorithm implemented in the Homology program [63] to maintain proper side-chain orientation. An energy minimization of the final models was carried out by 100 cycles of steepest descent using the cvff (consistent valence force field) forcefield of Discover and keeping the cysteine residues involved in disulphide bridges. The program Turbo-Frodo was run to draw the Ramachandran plot and to perform the superposition of the models. PROCHECK [64] was used to assess the geometric quality of the three-dimensional models. Molecular cartoons were drawn with PyMOL (W.L. DeLano, http://pymol.sourceforge.net).

### Surface Plasmon resonance analysis

SPR analysis was conducted on a Biacore X device (GE Healthcare, Saclay, France) set at a flow rate of 5μL/min using the CBEL glycoprotein purified from *P. parasitica* mycelium [18]. CBEL fixation on the sensor chip was achieved by hexadecyl-3-methylammonium bromide (CTAB) micelle-mediated immobilization under the following conditions: the sensor chip surface was first equilibrated in a 10 mM *N*-2-hydroxyethylpiperazine-*N’*-2-ethanesulfonic acid (HEPES) pH 7.4 buffer containing 1 mM CTAB. The sensor chip surface was then washed with 5 μl of 10 mM NaOH, and the carboxymethylated dextran sensor surface was activated by 35 μl of a mixture (1v/1v) of 100 mM *N*-hydroxy-succinimide and 400 mM *N*-ethyl-*N’*-(3-diethylaminopropyl) carbodimide. This activation was followed by injection of 80 μl of a solution of CBEL (12.5 μM) dissolved in 10 mM Hepes pH 7.4, 1 mM CTAB. Remaining ester groups were blocked by 35 μl of 100 mM ethanolamine chlorhydrate pH 8.5, before injection of 1 μl of 10 mM NaOH. Solutions of various pure standard glycoproteins dissolved in 10 mM HEPES pH 7.4, 150 mM NaCl, 3 mM EDTA, 0.005% vol/vol surfactant P20 (HBS-Ep buffer, Biacore) were then injected into the flow cell and passed over the CBEL surface during 1 min, and their interaction was followed in real time at different analyte concentrations. The tested glycoproteins were fetuin and asialofetuine (Sigma-Aldrich), human lactotransferrin (a kind gift of Dr H Debray, Université des Sciences et Technologies, Lille, France), and melon Hydroxyprolin-Rich GlycoProtein (HRGP; [43]).

Fetuin is a heavily *N*- and *O*-glycosylated protein where the most abundant *N*-linked glycans are triantennary species, and where both *N*- and *O*-linked glycans are sialylated on galactose terminal residues [65]. Human lactotransferrin glycans are of the sialyl *N*-acetyllactosaminic type and are fucosylated on *N*-acetylglucosamine residues [43]. HRGP is a *O*-glycosylated cell wall protein containing arabinose and oligoarabinoside side chains linked to hydroxyproline residues, and galactose units linked to serine residues [43,66]. Specificity of the CBEL-glycoprotein interactions was checked by measuring the level of protein complex dissociation in presence or absence of various monosaccharides. Complex dissociation was calculated at a fixed time point after injection during 5 minutes, at the beginning of the dissociation phase, of either HBS-Ep buffer or glucose, galactose or *N*-acetylgalactosamine (GalNAc) solutions. Data were analysed using the BIAviewer 3.1 software (Biacore AB, Uppsala, Sweden).

## Abbreviations

CBEL: Cellulose-binding elicitor lectin; CBM1: Carbohydrate-binding module, family 1.

## Competing interests

The authors are not aware of any financial, affiliations, funding that might be perceived as affecting the objectivity of this manuscript.

## Authors’ contributions

ML, RB, EG performed the computational analysis of the data. RB, BD, EG analyzed the computational results. ML and RB contributed equally to this work. AB conceived and performed the SPR analysis. A. Barre and PR carried out the 3D modeling of the CBEL protein. EG conceived the study and coordinated the manuscript. EG drafted and submitted the manuscript. All authors read and approved the final manuscript.

## Supplementary Material

Additional file 1 **Table S1.** List of fungal and oomycete species used in the current study. The phylogenetic classification of the species and the source of the data are indicated in the table. Click here for file

Additional file 2 **Table S2.** Alias used for domain organization of CBM1-containing proteins. The IPR number of individual domain appended to CBM1 is indicated, and the corresponding alias used to perform the heat-map of the distribution of architectures among fully sequenced fungi and oomycetes. Click here for file
