# Supplementary material for: The unique architecture and function of cellulose-interacting proteins in oomycetes revealed by genomic and structural analyses
Source: BMC Genomics. 2012 Nov 9;13:605. doi: 10.1186/1471-2164-13-605 (PMC3532174; doi:10.1186/1471-2164-13-605)
Supplement: Additional file 1 — Table S1. List of fungal and oomycete species used in the current study. The phylogenetic classification of the species and the source of the data are indicated in the table. [file 1471-2164-13-605-S1.pdf]

Supplemental table S1 : Sequenced eukaryote species used in the study

Name	Order	Data source
Albugo laibachii	Oomycota	EBI/ENA
Alternaria alternata	Ascomycota	CAZY
Aspergillus aculeatus	Ascomycota	CAZy
Aspergillus fumigatus	Ascomycota	CAZy
Aspergillus nidulans	Ascomycota	CAZy
Aspergillus niger	Ascomycota	CAZy
Aspergillus oryzae	Ascomycota	CAZy
Batrachochytrium dendrobatidis	Chytridiomycota	JGI
Blumeria graminis	Ascomycota	BluGenConsortium
Botrytis cinerea	Ascomycota	Broad Institute
Chaetomium globosum	Ascomycota	Broad Institute
Coccidioides immitis	Ascomycota	JGI
Cochliobolus heterostrophus	Ascomycota	CAZy
Colletotrichum graminicola	Ascomycota	Broad Institute
Colletotrichum higginsianum	Ascomycota	Broad Institute
Coniophora puteana	Ascomycota	CAZy
Coprinopsis cinerea	Basiodiomycota	CAZy
Cryphonectria parasitica	Ascomycota	JGI
Cryptococcus neoformans	Basiodiomycota	Broad Institute
Fusarium graminearum	Ascomycota	Broad Institute
Fusarium oxysporum sp. lycopersicum	Ascomycota	Broad Institute
Fusarium verticillioides	Ascomycota	Broad Institute
Glomus intraradices	Glomeromycota	CAZy
Heterobasidion annosum	Basiodiomycota	JGI
Hyaloperonospora parasitica	Oomycota	VBI Microbial DB
Hypocrea jecorina	Ascomycota	CAZy
Hypocrea virens	Ascomycota	CAZy
Laccaria bicolor	Basiodiomycota	JGI
Lactarius quietus	Basiodiomycota	CAZy
Leptosphaeria maculans	Ascomycota	JGI
Magnaporthe grisea	Ascomycota	CAZy
Malassezia globosa	Basiodiomycota	JGI
Melampsora populina	Basiodiomycota	JGI
Mucor circinelloides	Mucoromycota	CAZy
Mycosphaerella fijiensis	Ascomycota	CAZy
Mycosphaerella graminicola	Ascomycota	JGI
Nectria haematococca	Ascomycota	JGI
Neurospora crassa	Ascomycota	CAZy
Penicillium chrysogenum	Ascomycota	CAZy
Phanerochaete chrysosporium	Basiodiomycota	CAZy

Phytophthora capsici	Oomycota	JGI
Phytophthora infestans	Oomycota	Broad Institute
Phytophthora ramorum	Oomycota	JGI
Phytophthora sojae	Oomycota	JGI
Pichia pastoris	Ascomycota	CAZy
Pisolithus microcarpus	Basiodiomycota	Nancy
Pleurotus sp. Florida	Basiodiomycota	CAZy
Podospora anserina	Ascomycota	CAZy
Postia placenta	Basiodiomycota	JGI
Puccinia graminis	Basiodiomycota	Broad Institute
Pythium ultimum	Oomycota	Michigan State Univ
Rhizopus oryzae	Mucoromycota	CAZy
Schizophyllum commune	Basiodiomycota	JGI
Schizosaccharomyces pombe	Ascomycota	CAZy
Sclerotinia sclerotiorum	Ascomycota	Broad Institute
Serpula lacrymans	Ascomycota	JGI
Sporobolomyces roseus	Basiodiomycota	JGI
Sporisorium reilianum	Basiodiomycota	MIPS
Sporotrichum thermophile	Ascomycota	JGI
Stagonospora nodorum	Ascomycota	Broad Institute
Thielavia terrestris	Ascomycota	JGI
Trametes versicolor	Basiodiomycota	CAZy
Trichoderma viride	Ascomycota	CAZy
Tuber melanosporum	Ascomycota	TuberDB
Ustilago maydis	Basiodiomycota	Broad Institute
Verticillium albo-atrum	Ascomycota	Broad Institute
Verticillium dahliae	Ascomycota	Broad Institute
Arabidospis thaliana	Brassicae	TAIR
Oryza sativa	Funariaceae	Michigan State Univ
Porphyra purpurea	Rhodophyta	CAZy
Ectocarpus silicosus	Stramenopila	Bio Evol Gen
Phaeodactylum tricomutum	Stramenopila	JGI
Thalassiosira pseudonana	Stramenopila	JGI

Bio Evol Gen	http://bioinformatics.psb.ugent.be/genomes/view/Ectocarpus-siliculosus
BluGen Consortium	http://www.blugen.org
Broad Institute	http://www.broadinstitute.org/
CAZy	http://www.cazy.org/CBM1.html
EBI/ENA	http://www.ebi.ac.uk/ena/
JGI	http://genome.jgi-psf.org/
Michigan State Univ	http://pythium.plantbiology.msu.edu/
MIPS	http://mips.helmholtz-muenchen.de/genre/proj/sporisorium/

TAIR	http://www.arabidopsis.org
TuberDB	http://mycor.nancy.inra.fr/IMGC/TuberGenome/
VBI Microbial DB	http://vmd.vbi.vt.edu/
