# Supplementary material for: The unique architecture and function of cellulose-interacting proteins in oomycetes revealed by genomic and structural analyses
Source: BMC Genomics. 2012 Nov 9;13:605. doi: 10.1186/1471-2164-13-605 (PMC3532174; doi:10.1186/1471-2164-13-605)
Supplement: Additional file 2 — Table S2. Alias used for domain organization of CBM1-containing proteins. The IPR number of individual domain appended to CBM1 is indicated, and the corresponding alias used to perform the heat-map of the distribution of architectures among fully sequenced fungi and oomycetes. [file 1471-2164-13-605-S2.pdf]

Supplemental table S2 : Alias used for domain organization of CBM1-proteins. The Pfam/Smart/InterPro (IPR) accessions number of individual domain appended to CBM1, and the corresponding alias used to perform the heat-map of the distribution of architectures among fully sequenced fungi and oomycetes are presented in the table.

Name	PFAM/SMART	IPR		Alias
**Carbohydrate Binding Module (CBM)**				
Family 1	PF00734	IPR000254		CBM1
Family 6	PF03422	IPR005084		CBM6
**Glycoside Hydrolase**				
Glycoside hydrolase, family 3	PF01915	IPR002772	IPR001764	GH3
Glycoside hydrolase, family 5	PF00150	IPR001547		GH5
1, 4-beta cellobiohydrolase	PF01341	IPR016288		GH6
Glycoside hydrolase, family 7	PF00840	IPR001722		GH7
Glycoside hydrolase, family 10	PF00331	IPR001000		GH10
Glycoside hydrolase, family 11	PF00457	IPR001137		GH11
Glycoside hydrolase, family 12	PF01670	IPR002594		GH12
Glycoside hydrolase, family 17	PF00332	IPR000490		GH17
Glycoside hydrolase, family 18	PF00704	IPR001223		GH18
Glycoside hydrolase family 27/36	PF02065	IPR000111		GH27/36
Glycoside hydrolase, family 43	PF04616	IPR006710		GH43
Glycoside hydrolase, family 45	PF02015	IPR000334		GH45
Alpha-L-arabinofuranosidase B	PF09206	IPR015289		GH54
Glycoside hydrolase, family 61	PF03443	IPR005103		GH61
Glycoside hydrolase, family 62	PF03664	IPR005193		GH62
**Oxydoreductase, Lyase, Esterase**				
Glucose-methanol-choline oxidoreductase	PF05199	IPR007867	IPR000172	OXY
Pectate lyase/Amb allergen	PF00544	IPR002022		LYA
Pectate lyase, catalytic	PF03211	IPR004898		LYA
Parallel beta-helix repeat	SM00710	IPR006626		LYA
Cutinase	PF01083	IPR000675		EST
Lipase, GDSL	PF00657	IPR001087		EST
Phospholipase/carboxylesterase	PF02230	IPR003140		EST
Peptidase S9, prolyl oligopeptidase	PF00326	IPR001375		EST
Esterase_phd	PF10503			EST
**Misceallous**				
BNR repeat	PF02012	IPR002860		BNR
Apple + PAN-1 domain	PF00024/SM00223	IPR000177	IPR003014	PAN
Chitin-binding, type 1	PF00187	IPR001002		CHI
Fungal fucose-specific lectin	PF07938	IPR012475		FUC
Pollen allergen/expansin	PF01357	IPR007117		EXP
DUF		IPR005102	IPR018789	DUF
		IPR021840	IPR018535	

** InterPro accession number*

*** alias used during the study*
